# An ontology network for Diabetes Mellitus in Mexico

**DOI:** 10.1186/s13326-021-00252-2

**Published:** 2021-10-09

**Authors:** Cecilia Reyes-Peña, Mireya Tovar, Maricela Bravo, Regina Motz

**Affiliations:** 1 Faculty of Computer Science, Benemerita Universidad Autonoma de Puebla, Av. San Claudio, Puebla, Mexico; 2 Universidad Autonoma Metropolitana, Av. San Pablo No. 180, Mexico City, Mexico; 3 Universidad de la Republica, Julio Herrera y Reissig 565, Montevideo, Uruguay

**Keywords:** Knowledge representation, Ontology network, Design methodology

## Abstract

**Background:**

Medical experts in the domain of Diabetes Mellitus (DM) acquire specific knowledge from diabetic patients through monitoring and interaction. This allows them to know the disease and information about other conditions or comorbidities, treatments, and typical consequences of the Mexican population. This indicates that an expert in a domain knows technical information about the domain and contextual factors that interact with it in the real world, contributing to new knowledge generation. For capturing and managing information about the DM, it is necessary to design and implement techniques and methods that allow: determining the most relevant conceptual dimensions and their correct organization, the integration of existing medical and clinical information from different resources, and the generation of structures that represent the deduction process of the doctor. An Ontology Network is a collection of ontologies of diverse knowledge domains which can be interconnected by meta-relations. This article describes an Ontology Network for representing DM in Mexico, designed by a proposed methodology. The information used for Ontology Network building include the ontological resource reuse and non-ontological resource transformation for ontology design and ontology extending by natural language processing techniques. These are medical information extracted from vocabularies, taxonomies, medical dictionaries, ontologies, among others. Additionally, a set of semantic rules has been defined within the Ontology Network to derive new knowledge.

**Results:**

An Ontology Network for DM in Mexico has been built from six well-defined domains, resulting in new classes, using ontological and non-ontological resources to offer a semantic structure for assisting in the medical diagnosis process. The network comprises 1367 classes, 20 object properties, 63 data properties, and 4268 individuals from seven different ontologies. Ontology Network evaluation was carried out by verifying the purpose for its design and some quality criteria.

**Conclusions:**

The composition of the Ontology Network offers a set of well-defined ontological modules facilitating the reuse of one or more of them. The inclusion of international vocabularies as SNOMED CT or ICD-10 reinforces the representation by international standards. It increases the semantic interoperability of the network, providing the opportunity to integrate other ontologies with the same vocabularies. The ontology network design methodology offers a guide for ontology developers about how to use ontological and non-ontological resources in order to exploit the maximum of information and knowledge from a set of domains that share or not information.

## Background

There are currently many vocabularies with medical and scientific terminology available on the Internet. However, not all the information has a clear structure; for example, with the COVID-19 pandemic, the consumption of medical knowledge has increased around the symptoms and relations with other diseases. According to a study on the Mexican population [1], obesity, Diabetes Mellitus (DM), and Hypertension were significantly associated with severe COVID-19.

DM is a metabolism alteration that occurs due to increased glucose levels in the blood related to insulin production in the body. The most common types of DM are Type 1 Diabetes Mellitus (T1DM) and Type 2 Diabetes Mellitus (T2DM). However, there are other types of DM, such as gestational diabetes, caused by genetic defects, infections, among other factors [2]. In Mexico, for every 100,000 inhabitants, 339.39 are diabetic patients [3]; however, there is a lack of information within the population about the prevention, symptoms, treatment, and types of DM, which can generate significant health problems due to postponing a timely medical check-up.

Currently, medical experts in the DM domain acquire knowledge of the disease through monitoring and interaction with the patient, which allows them to know the disease and information about other conditions or comorbidities, treatments, and some consequences, typical of the conditions of the Mexican population. The above indicates that an expert in a domain knows technical information about the domain, contextual factors, and the interactions with the real world that contribute to new knowledge generation. For capturing and managing information about the DM, it is necessary to design and implement techniques and methods that allow: determining the most relevant conceptual dimensions and their correct organization, the integration of existing medical and clinical information from different resources, and the generation of structures that represent the deduction process of the doctor.

It is necessary to consider that these tools should provide legible information representation models that facilitate medical experts to make clinical decisions based on data, symptoms, and diseases from the diabetic patient. One option that covers these requirements are the ontologies.

An ontology is a representation of a part of the real world; this part is known as a domain. Formally, Gruber [4] defined *An ontology is an explicit specification of a conceptualization*; this definition is complemented in [5, 6] explaining that an ontology is a *representational vocabulary for a shared domain of discourse* defined through classes, relations (properties), functions, individuals, and semantic rules; where each ontology and each element within the ontology are identified by a unique IRI (Internationalized Resource Identifier). Ontologies are a good option for the construction of semantic models. They allow the inclusion of human-machine readable information, definitions from logical description predicates, and reasoning by information from medical records and axioms. It will facilitate the discovery of knowledge that enriches the risk factor identification for DM, its relation with other possible diseases, and its treatment.

Ontologies in the medical domain are very useful since they allow the exchange of information acquired from various sources such as books, articles, experience, and knowledge from experts in an organized and legible way. Currently, medical ontologies are using to improve the literature and teaching and generate new information through inference.

There are several ontologies about DM diagnosis [7–12], DM consequences [7, 11, 13–17], about DM care and diabetic lifestyles [14, 18, 19], and diabetic patient electronic records management and monitoring [20–22]. Table 1 lists the main features of these DM ontologies relating to the content and use of ontological elements (such as semantic rules and ontology evaluation), which reflect some of the general considerations of DM ontologies. Although each of them contains relevant information about domains that directly impact diabetes, they lack additional information about the domains. This limits the design of a complete ontological model that can support decision-making in cases of diabetic patients who suffer additional diseases or who have been prescribed pharmaceutical treatments in addition to those to DM control. Some domains that can enrich the DM ontologies are vocabularies about disease classification [23–26], drug classification and their interactions [27–31].

**Table 1:** Existing ontology for DM

Cite	T1DM	T2DM	FH	PD	Tt	DDR	SR	Observations	Evaluation
[7]	×	$\checkmark $	×	$\checkmark $	×	$\checkmark $	×	This system starts from variables to determine in a diffuse way if a patient suffers from diabetes or the risk of suffering from it.	Performance comparison facing Machine Learning-based classifiers.
[8]	$\checkmark $	$\checkmark $	×	$\checkmark $	×	×	$\checkmark $	This ontology proposes the degree of suffering from different types of diabetes based on specific characteristics presented at a given time in a patient.	Not indicated.
[9]	×	$\checkmark $	$\checkmark $	$\checkmark $	×	$\checkmark $	$\checkmark $	The relation between diabetes with other diseases is only used to diagnose diabetes, not to suggest a future condition.	Completeness, abstraction, cohesion, conceptualization, complexity and understanding.
[10, 11]	×	$\checkmark $	$\checkmark $	$\checkmark $	$\checkmark $	$\checkmark $	$\checkmark $	Does not include insulin-based treatments or their consequences.	Validation and verification.
[12]	×	$\checkmark $	×	$\checkmark $	×	×	$\checkmark $	This ontology is limited to proposing a degree of propensity to suffer from diabetes and does not include any additional consequences.	Consistency by Kappa Index.
[13]	×	$\checkmark $	×	×	×	$\checkmark $	×	This ontology represents knowledge associated with diabetes, limiting itself to containing only classes and relation based on the information represented in other ontologies.	Validation by domain experts.
[14]	×	$\checkmark $	×	$\checkmark $	×	$\checkmark $	$\checkmark $	This ontology proposes the degree of suffering from diabetes and only includes heart conditions as a consequence of it.	Functionality, reliability, efficiency, maintainability and portability.
[15]	×	$\checkmark $	×	$\checkmark $	$\checkmark $	$\checkmark $	$\checkmark $	This model does not provide a diagnosis of possible diseases, it only provides established information about life styles for diabetic patients.	Accuracy and consistency.
[16]	×	$\checkmark $	×	$\checkmark $	×	$\checkmark $	$\checkmark $	This ontology proposes the degree of complications from values related to laboratory tests, age, and obesity degree, among others.	Consistency.
[17]	$\checkmark $	$\checkmark $	×	$\checkmark $	×	×	$\checkmark $	This system is limited to working only with signs that occur in a person at a specific time, regardless of their clinical history.	Cases of use.
[18]	$\checkmark $	$\checkmark $	×	$\checkmark $	$\checkmark $	×	$\checkmark $	This ontology has the purpose of informing about the lifestyle of a diabetic patient, discriminating the diagnosis of the disease.	Scenarios and consistency.
[19]	×	$\checkmark $	×	×	$\checkmark $	$\checkmark $	$\checkmark $	This ontology represents knowledge associated with the care of diabetic patients, so it does not include any type of diagnosis.	Not indicated.
[20]	×	$\checkmark $	$\checkmark $	$\checkmark $	$\checkmark $	×	×	This model is only designed to classify files based on structured information, making it impossible to give a diagnosis.	Not indicated.
[21]	×	$\checkmark $	×	$\checkmark $	×	×	$\checkmark $	This proposal is limited to working only with clinical records, so it is incomplete as it does not have knowledge from a specialist doctor.	Not indicated.
[22]	×	$\checkmark $	×	$\checkmark $	$\checkmark $	×	$\checkmark $	This ontology contains knowledge associated with diabetes, limiting itself to providing medication and food suggestions based on a patient’s condition.	Recall, Precision, Accuracy and F-Measure.

Abbreviations. *FH* Family History, *PD* Personal Data, *Tt* Treatment, *DDR* DM-Disease Relation, *SR* Semantic Rules

Existing ontologies can be reused to expand the representation of the domain or avoid building them from scratch and thus save resources. In the case of expanding a representation, there are two cases in general that can be presented: ontologies have semantic correspondences (elements with the same meaning) about same domain or ontologies do not have semantic correspondences. So in the first case, an ontology can be constructed using their correspondences by Mapping [32, 33] or Alignment [32, 34]. In contrast, in the second case, when ontologies do not have semantic correspondences among them, it is possible to construct an ontological model integrating them; and by adding additional information, this model can become an Ontology Network.

Unlike ontology integration tasks such as Mapping or Alignment, Ontology Networks involve ontologies that may or may not have semantic correspondences between them and are defined as a collection of ontologies related by meta-relations that indicate the dependency between them [34]. In view of the above, the Ontology Network design refers to the creation of meta-data between classes from different ontologies in order to create a new ontological model.

The meta-relations in an Ontology Network are defined from the participation of the ontologies used in the network [35] and are independent of the represented domains in the ontologies [36]. Since ontology networks are a collection of ontologies, it is crucial to consider the number of computational resources required to reason about them and query their individuals. Therefore, it is important to consider the expressiveness of reuse and design ontologies since high expressiveness requires high computational resources.

In the literature, there is a methodology for Ontology Network construction, which is based on scenarios focused on guiding the use of each of the available resources [37]. Also, there is software for the same purpose, which uses only existing ontologies [38]. However, none presents a guide for incorporating non-ontological resources such as information written in natural language from medical records without previously building an ontology that represents them.

Given the above, the methodology proposed in this paper addresses the problem of capturing and managing information about the prevention and diagnosis of DM. It requires the design and implementation of an Ontology Network that allows the use of existing medical knowledge in vocabularies, taxonomies, medical dictionaries, ontologies, non-ontological resources, and the generation of rules that represent the deduction process of the medical expert.

This article describes the design methodology used for the Ontology Network construction of DM in Mexico by the ontological resource reuse and non-ontological resource transformation for ontology design and ontology extending by natural language processing techniques. The used resources are medical information extracted from vocabularies, taxonomies, medical dictionaries, ontologies, among others. Additionally, a set of semantic rules has been defined within the Ontology Network to derive new knowledge.

The ontology network approach was chosen because having a better notion of each of the domains necessary for the representation of the DM and thus achieving the critical elements of each one to create a lightweight ontological model using only the needed information. Another advantage of using this approach is exploiting the information from non-ontological resources to enrich one or more ontologies simultaneously. In addition, maintenance tasks such as analysis of the causes of change, effects of different operations on the data, and creating different versions [39], can be performed on each of the participating ontologies within the network without altering the ontologies or compromising the consistency of the network, allowing to make the ontology network functional despite the evolution of its components.

The remainder of this paper is organized as follows. The “Methods” section shows the design methodology used for Ontology Network building; the “Results” section contains the relevant features and the evaluation of the Ontology Network. “Discussion” section presents a review of the applied methodology regarding the obtained results; and finally, the “Conclusions” are expressed in the last section.

## Methods

This section describes the construction of the Ontology Network for DM, considering the features of the Mexican population, DM treatments, DM-Diseases relation, among other aspects, using an Ontology Network Design Methodology. The following subsections describe the methodology steps, starting from the definition of the elements of the network, the participating domains, including their scope; subsequently, the search and acquisition of the necessary resources for each domain, as well as a set of ontological engineering tasks for the design, reuse, population, and evaluation focused on the concept of Ontology Networks.

### Step 1: Relevant definitions

In this step, the relevant definitions mark the features that the ontology network design must have been established, mainly determining the purpose and scope.

#### Ontology network requirements

El-Sappagh et al. [11] suggest that in order to define the domain and the requirements, the following question should be answered: *What part of the real world corresponds to Ontology Network?*. In this work, the part of the real world corresponds to the task of medical diagnosis related specifically to DM, its treatment, and its possible consequences, considering the demographic and clinical elements of the Mexican population. For this, domains and their features can be defined through competency questions considering the possible usage scenarios and the end-users [11, 40, 41]. The competency questions are:

1. What are the most common demographic features related to diabetic patients?
2. What are the most common complications related to DM?
3. What are the pharmacologic treatments most recommended for diabetic patients?
4. What are the data inserted into the clinical record of a diabetic patient?
5. What is the human biotype of a patient weighing *X* kg and is *Y* meters tall?
6. What are the values of vital signs of a diabetic patient?
7. What are the amount of active pharmaceutical ingredients *X* recommend for the patient *Y*?

In order to identify the main domains, it is necessary to apply a Term elicitation technique like the one proposed in [41], where the nouns are extracted from the competency question as Disease, Diabetic Patient, Treatment, Clinical Record, and Demographic Feature; these terms represent each of the main domains of the network. Once the main domains have been identified, a more detailed analysis is needed based on the general requirements in order to derive the specific requirements of each domain. Proposed examples for generating new competency questions that can be answered with information from a single domain, are shown below:

- Diabetic Patient:
  1. What is the weight of a patient?
  2. How many siblings do a patient have?
  3. What is the body mass index of a patient?
  4. What was the highest amount of glucose in the blood that a patient had?
  5. What is the patient’s age?
  6. What is the sex of the patient?
- Disease:
  1. What diseases can be the consequence of T2DM?
  2. What is the ICD-10 code for T2DM?
  3. What are the comorbidities that may be present in a diabetic patient?
  4. What are the disabilities that may be present in a diabetic patient?
  5. What are the most frequent diagnoses of patients with DM?
  6. What is the SNOMED CT identifier for insulin-dependent DM?
- Treatment:
  1. What are the types of treatment recommended for diabetic patients?
  2. What are the hypoglycemic drugs that have an oral route of administration?
  3. What are the presentations of Insulin Lispro?
  4. What are the medications prescribed for diabetic patients?
  5. What is the risk during the pregnancy of human insulin?
  6. What are the recommended doses of Metformin?
- Demographic Features:
  1. Where the patient *X* is living?
  2. What is the education level of the patient *X*?
  3. What are the non-pathological history of the patient *X*?

Finally, the expected features per domain that the network should have been listed below. The demographic domain is divided into specific domains as Education Level and Geographic Location.

- Patient: it should describe what a person is and the relation with information on signs, inherited-family history, and personal data such as weight, height, age, among others.
- Clinical Entity: represents the diseases and symptoms that may appear in the diabetic patient’s diagnoses, including disabilities or comorbidities, as well as the relation with some symptoms and other consequent diseases.
- Control Plan: mainly represents the pharmaceutical treatments suggested for the DM diagnosis and additional information on the drugs.
- Education Level: it contains the different education levels in Mexico.
- Geographic Location: describes the political division of the Mexican territory.
- Clinical Information Administration: this must store and manage clinical information as the clinical record. It has an essential role in the network because it connects the different domains through their content. The content must be associated with the date on which the update of a patient’s data is presented during the clinical consultation, such as analysis results, weight, diagnoses, and treatments, among others. Liaw et al. [20] developed an ontology for the management of clinical records from a database since it is important to design an ontology that models the clinical record in order to include the variety of formats of clinical record within the Mexican health system.

#### Data sources

The proposed strategy for acquiring medical data starts with searching for information on the medical domain contained in both ontological and non-ontological resources. Some examples of these resources are books, websites, catalogs, ontologies, vocabularies, among others. Resources that do not provide information on the topics in the ontology network (patient, disease, medical history, and treatment) must be discarded.

The search for ontological resources should focus on obtaining ontologies for each domain and satisfying the requirements defined in the ontology network for each determined domain in the first stage of the methodology. For disease representation, there is SNOMED CT [24], which offers a broad clinical terminology about various domains such as diseases, demographic information, some pharmacological treatments, among others. However, the granularity used in SNOMED CT is very detailed and does not match with the terminology used in the public Mexican health system. DO [42] is an ontology of another disease classification; its classification does not match with the regulations of the secretary of health about the use of the ICD-10 code. ICDO [43] offers an ontology about the ICD-10 classification, within which there is little readable information about the classification. For drug representation, there is an ontology from a part of drug catalog edition 2017 (Drug Ontology [44]) and contains the terminology used by the medical centers that are belonging to the Mexican system.

For the drug domain, there is the Basic Table and Catalog of Drugs edition 2017, which contains the pharmacologic treatment approved for use and distribution by the public Mexican health system approved by of Secretary of Health from Mexico and available in its portal [45]. The catalog structure is divided in two elements: a table with the identifier, description, indications, and doses and route of administration; and the generalities, secondary effects, interactions, cautions and contraindications. The last elements are expressed in natural language, while the first elements are represented in the Drug ontology [44].

To start collecting non-ontological resources, the portal of the Secretary of Health of Mexico was selected, where the Epidemiological DM Bulletins [46] are found. These bulletins contain the statistics on the main characteristics of the Mexican population related to DM registered in hospitalization. Also, in the same portal, it was possible to find some specifications about the content of clinical records as the use of the International Classification of Disease (ICD-10).

One of the most important resources is clinical records composed of one clinical history and one or more medical notes. Each clinical history contains information about Hereditary-Family, Gyneco-Obstetric, Pathological and Non-Pathological histories. The medical note includes data about the signs and symptoms, weight, height, glucose values, and a description of physical examination, among others. In addition, the medical note contains a prescription according to one or more diseases indicated as medical diagnoses. For the acquisition of clinical data, 171 clinical records were captured and provided by the University Hospital^1^. Of the 171 clinical records, 90 belong to male patients and 81 to female patients. There are a total of 729 diagnoses from medical notes, of which 143 correspond to insulin-dependent diabetes, 149 to non-insulin-dependent diabetes, and 437 to other clinical entities; in terms of medical treatment, there are a total of 1626 prescribed medications, of which Insulin Glargine is the most prescribed with 131 recommendations, Metformin with 61 and 58 Diosmin with Hesperidin.

#### Analysis of the resources obtained

At this stage, two cases are presented to address each domain representation according to the resources obtained in the previous stage. One uses non-ontological resources to create an ontology from scratch, prioritizing specific information. The other is to use ontologies already established to form or enrich the network. For this, it is necessary to identify the structure of the information and evaluate whether creating a new ontology is pertinent or can be used to design the meta-relations of the network.

For DM Ontology Network design, three critical resources were acquired: epidemiological bulletins of T2DM of the last five years to identify the most common features of the Mexican population related to this disease, the 326 medical records of diabetic patients, and the catalog of medications of the secretary of health. The information structure of each are described below:

1. Epidemiological bulletins: these contain statistical information about demographic and clinical features of the Mexican population as the distribution of T2DM cases by state, age group and sex, education level, human biotype, and according to a type of family history; the comorbidities and disabilities present in diabetic patients; the control plans indicated for patients with T2DM, the proportion of the type of intra-hospital insulin indicated for the control, and the type of care service for patients with T2DM.
2. Medical Records: Medical records contain a set of medical notes and patient data sheets regarding patient history as part of the medical record.
  Medical history: Personal pathological history, Non-personal pathological history, Hereditary-family history, Gyneco-Obstetric History, and Allergies.
  Medical note: Date, Current condition, Physical examination, Diagnosis, Management plan, Treatment, and Forecast.
3. Drug catalog: contains information about the drugs authorized and distributed by the Secretary of Health of Mexico.

Once the relevant concepts were identified, the ontologies that would serve as the basis for the integration of the different domains that participate in the ontology network were built.

### Step 2: New ontology design

This stage addresses ontology design from scratch. The most appropriate design methodology can be worked according to the nature of the information structure or automating tasks of each stage according to its complexity.

**Fig. 1:**
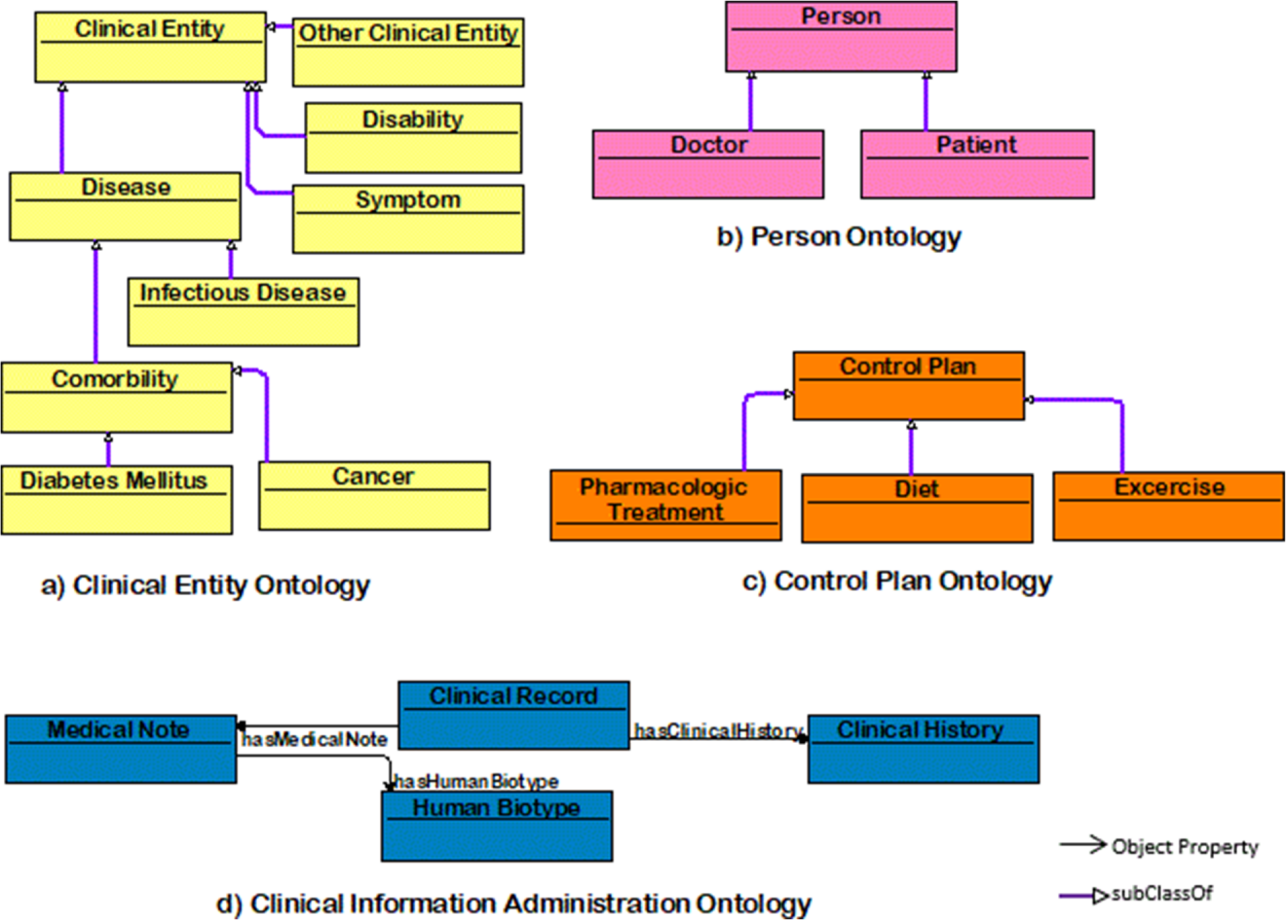
Designed Ontologies. Some of designed from scratch ontologies

Subsequently, new ontologies were built using only the statistical information from the bulletins as a reference for incorporating demographic information on the Mexican population into the DM Ontology Network. These are specific ontologies, without many concepts involved, but considering them as the basis of the network, we carry out a consistency evaluation on them. Given the above, a total of six domain ontologies were created from scratch, some of them can be seen in Fig. 1, and the general description is detailed in Table 2. Each ontology contains individuals according to its domain and may contain some data-types and object properties within itself.

**Table 2:** Ontologies per domain

Ontology Name	Domain Description	Ontology IRI
Control Plan	Contains the control plan recommended for a disease considering diets, exercise, and pharmaceutical treatment.	http://www.medida-control.com/tratamiento
Clinical Entity	About a classification of disease, comorbidities, and disabilities.	http://www.padecimientos-mexico.org/enfermedades
Education Level	Contains the academic level classification according to Mexico’s structure.	http://www.niveleseducativos-mexico.org/niveles
Clinical Information Administration	Represents the management of information about clinical data as clinical records, medical notes, and medical histories.	http://www.modelo.org/datos
Geographic Location	Contains the 32 states from Mexico.	http://www.estados-mexico.org/estados
Person	About the classification of person roles as patients and doctors considering the data of each one.	http://www.personas-mexico.org/persona

### Step 3: Ontology reuse

Ontology Reuse in an ontology network refers to taking an existing ontology and integrating it into the network, partially or completely, to expand or specify the domain of the ontology network. It allows to lower the cost of design against the design of ontologies from scratch; however, several considerations must be taken into account when reusing ontologies within an Ontology Network, such as:

- The ontology covers the domain requirements to participate in the network. Evaluate whether the enrichment of the model can solve the lack of information and determining if there are elements that are not necessary for the network. In this regard, it might sound attractive to preserve information not considered in the definition of the network. However, verifying whether the model’s size is optimal is essential to avoid the high demand for computational resources to execute inferences with the network. If the request is high, an alternative would be to apply some modularization method to keep only the relevant information.
- The ontology fully covers domain requirements to participate in the network, and the information granularity from both matches the specifications of the network.

Once the situation of the ontology to be reused is clear, it should be verified if there are any correspondences between two participating ontologies of the network [47]. If there are, a decision must be made between using a traditional integration methodology or the Mapping and Alignment tasks, where both ontologies will be imported into the network; otherwise, using an integration methodology through external references.

#### Ontology integration

One of the main problems for ontology integration is the diversity of the representations of the elements. For example, synonyms, which implies representing the same thing with different names; homonyms, elements that have the same names, but their meaning is different; and concepts in the ontologies that are correspond in different ways [48]. Not having a well-defined strategy for integrating ontologies can lead to inconsistency problems that can alter the operation of ontologies [49]. For addressing this problem, an ontology integration methodology has been proposed and applied for the Drug Ontology and ATC ontology to facilitate the reusing of the Mexican catalog drugs by integrating an international standard. The stages are described below:

1. Determine the base ontology to enrich: select a base ontology that will be enriched in order the resulting ontology does not lose the functionality of fulfilling the task for which it was originally designed. In this stage, the Drug Ontology was taken as the base since its features satisfy the drug domain requirements necessary for the ontology network.
2. Identify elements with the same names: find terms of the ontology to be integrated that have the same name with respect to the elements of the base ontology. Synonymous terms must also be considered; for this, it is necessary to rely on additional resources (dictionaries, Thesauri, domain literature, among others). So, when reviewing the ATC Ontology, it found coincidences in the names of the active pharmaceutical ingredients at the lowest levels of the class hierarchy and those used in the instances of the belonging active pharmaceutical ingredient class to the Drug ontology.
3. Semantic Verification: it must be verified if the terms found in the previous stage represent the same entities. It is recommended to use appropriate terminology for the representation of the terms. Subsequently, by searching for more references in external resources such as web pages and documents about the pharmacologic elements, the semantic coincidence can be confirmed.
4. Analyze how the common elements are represented (classes, instances, and properties): in order to define a way in which there are more benefits than the others. One way to represent the common elements from the Drug and ATC ontologies is to keep the taxonomy of ATC ontology. However, there would be redundancy since existing classes and instances represent the same thing. Another way is to convert the lower levels of the taxonomy into instances of the immediate superior class, keeping the key as an identifier and the name as a data type property; despite this, there would still be redundancy between instances that would have the same name with a different key. Considering the pros- and cons- of the alternative representation of the common elements, we concluded that the best option is to convert the lowest classification levels to instances and to instance them from the immediate superior class. The key of each instance must be replaced by a data-type property with a unique value allowing the instance name to be used as a unique identifier.
5. Determine the final structure: redefine the correspondences found in the common elements to preserve the consistency of both ontologies. In this step, new elements will be created to complement the representation if necessary. When observing that the Drug ontology only associated one active pharmaceutical ingredient per drug, despite there are drugs that have a combination of two or more of them, it is necessary to create the *Active Pharmaceutical Ingredient Mapper* class, which contains anonymous nodes with the *hasAmountOfActivePharmaceuticalIngredient* data property. The class serves as an intermediary of the object properties *hasActivePharmacueticalIngredientPerPortion* and *hasActivePharmaceuticalIngredient* (this object property has as range the ATC Classification class) since it provides an attribute value for each relation. Through this change, the *Active_Pharmaceutical_Ingredient* class of the drug ontology is eliminated since it only had individuals of the names of active pharmaceutical ingredients and did not contain any additional information.
6. Resulting Ontology Evaluation: for a first evaluation of the new integrated ontology, it is necessary to verify the fulfillment of the original purpose for which the ontology was designed before being integrated. Subsequently, the benefits of the integrated ontology should be highlighted through new competency questions that represent its new use cases. Finally, for evaluating the resulting ontology, the consistency criterion was checked, and the competency questions of the drug ontology design in [44] were answered again to ensure that it continues to fulfill the original purpose.

**Fig. 2:**
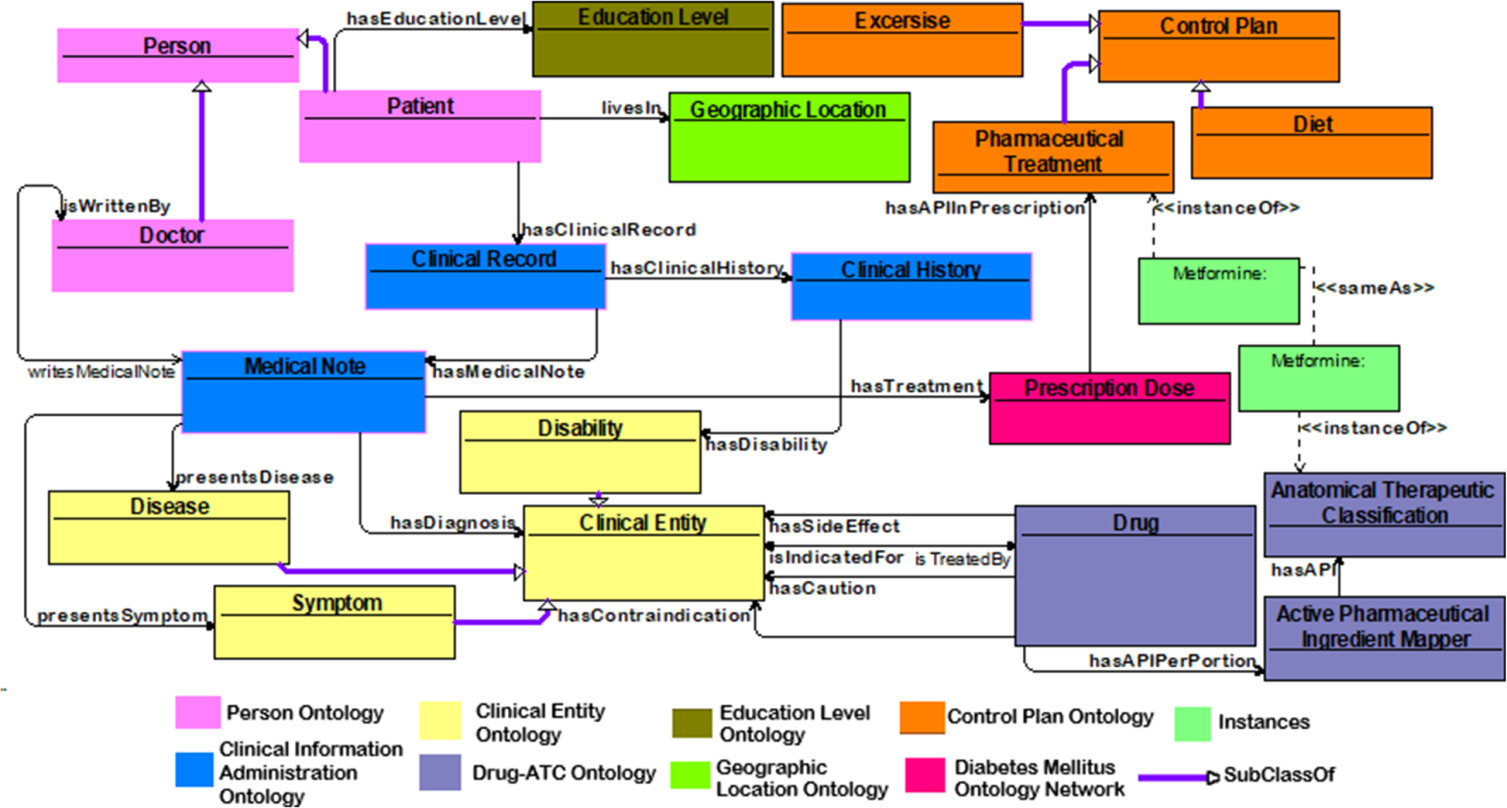
DM Ontology Network Diagram. The diagram contains some of all classes that composed the network in order to show the use of meta-relations as object properties

As a result of the integration, the Drug-ATC ontology has modifications. The *Active_Pharmaceutical_Ingredient* class was replaced by the connection of the anonymous nodes, which have quantity and measure whose values depend on the instances to which they are related. The ontology evaluation through DL Expressivity continues to be maintained in ALCQ(D), and the competency questions related to the modified elements were answered again. Subsequently, the Drug-ATC Ontology is integrated with the ontology generated from the information in the bulletins about the Control Plan, having as clear correspondences the individuals of the active pharmaceutical ingredients, for which a set of *sameAs* object properties are established between both elements. Figure 2 shows an example about the Metformin, it is an instance of Drug ontology, and there is an instance with the same name belonging to Control Plan ontology, both keep in the ontology network and were associated by the *sameAs* property.

#### External reference method

The purpose of the external reference method is to integrate relevant information about other ontologies or vocabularies into an ontology through attributes without the need to import the complete information source. This method is useful when the referenced information will only be used through queries because if the final application of the ontology requires reasoning tasks, this method is not correct due to the information restriction. The steps for the external reference method are:

1. Identification of the vocabulary and its reference structure: define where the information will be taken from and what elements may be key to referring. It is important to use an attribute whose value is unique or function as an identifier when using ontological resources.
2. Analyze how the elements will be integrated into the ontological model: in this step, it must define how the reference selected in the previous step will be integrated. It must consider that this form must be provided to create a possible link to the original information source, perhaps through an application of ontology. This step must also consider what elements the class or instance the reference will be assigned to.
3. Format query in application: define if direct reference links will be created from the ontology or network application, or if they will only be displayed as a result of a query.

As a result of applying the external reference method, we select SNOMED CT as the reference for Disease ontology. Although the granularity does not coincide with that used by the health system in Mexico, there are some common elements. These can be used to create a link that the users can follow if they want to find more information about those elements. In addition, by taking SNOMED CT references, the final Clinical Entity ontology increases its level of interoperability because SNOMED CT is an internationally recognized vocabulary. For this, only the SCTID is considered Annotation Property for the diseases; in this way, we can reduce the demand for computational resources by not including the rest of the structure. Another considered ontology is ICD-10 which complies with the guidelines established by the Mexican Secretary of Health.

### Step 4: Meta-relation design

The meta-relation design is one of the essential parts of the ontology network design since they are in charge of interconnecting the different domains, giving real meaning to the ontology network. This step proposes to work in pairs of ontologies (*A*, *B*). Also, it must have information about how each of the components of the ontology *A* interact with the ontology *B* components.

Once the interactions have been identified, the next step is to define which interactions are relevant for the ontology network and discard the rest. Otherwise, the computational requirements of the network will overgrow. Also, it should consider whether ontologies that have a strong dependency between them (such as versioning) will participate within the network. If necessary, the dependencies should be regarded as meta-relations. Then, when the interactions have been selected, they must be adapted to the terminology of a traditional ontology: select a conjugated verb in the third person for the property’s name, establish the domain and range, and implement the necessary cardinality restrictions.

All meta-relations will have an IRI independent from those established within the base ontologies, taking the IRI from the ontology network. Table 3 shows the possible correspondence of each of the classes by ontology, a selection criterion of meta-relations could be to avoid redundancy; for example, the meta-relations Patient-hasClinicalRecord-Clinical Record and Patient-hasMedicalNote-Medical Note. When analyzing the structure of the Clinical Information Ontology, it can observe that exists the object property Clinical Record-hasMedicalNote-Medical Note. So it is more convenient to keep the meta-relation Patient-hasClinicalRecord-Clinical Record. This concludes that the patient having a clinical record will also have a set of medical notes to be more reliably attached to the real world. Another example is the case of pharmacological treatment and diagnosis. Although a patient may have one, the correct thing to do is to handle it in the medical note since these relations may vary according to the time of registration because using them within the medical note would allow keeping those records associated with a date.

**Table 3:** Candidate meta-relations

Domain	Candidate Meta-relation	Range
Patient	hasClinicalRecord	Clinical Record
Patient	hasMedicalNote	Medical Note
Patient	hasTreatment	Prescription Dose
Person	livesIn	Geographic Location
Person	hasEducationLevel	Education Level
Person	hasDiagnosis	Disease
Medical Note	hasTreatment	Prescription Dose
Medical Note	hasDiagnosis	Disease
Drug	isIndicatedFor	Disease
Medical Nota	isWrittenBy	Doctor
Doctor	writes	Medical Note
Doctor	attends	Patient
Patient	isAttendedBy	Doctor

### Step 5: Non-ontological resources integration

The integration of non-ontological resources into an Ontology Network can enrich the representation in different ways: facilitating the identification of meta-relationships, as well as the population with individuals that satisfy the meta-relationships.

In the following, the stages of a methodology for integrating non-ontological resources within an existing ontology are described. They are focused on suggesting triples that could be relevant within the domain by task from the requirement analysis and the purpose identification, and the evaluation of the results.

#### Purpose and information features identification

For starting this methodology, it is necessary to be clear about the need to be satisfied through an ontological representation and identify why an existing ontology does not satisfy this need by itself.

Once the purpose has been identified, the resources involved must be selected, that is, determining an existing ontology that can provide a partial solution and the non-ontological resources to integrate that can be compatible with the ontology in order to provide a complete solution together.

If the integration of non-ontological resources does not require the ontology population, the next stage can be omitted.

#### Ontology population

In the ontology population process, it is necessary to identify the individuals and their properties within the corresponding parts of the network.

For the DM Ontology Network, the non-ontological resources from the medical notes are used as instances of each ontology. Each instance of Person class must have a relation towards Clinical Record that contains one or more instances of the Medical Note class. The Diagnosis section within the medical notes includes diseases that are instances of Disease class; also, many parts of medical notes contain values associated with the data-type properties established in the Clinical Information Administration ontology.

#### Candidate element identification and their ontological form selection

The candidate elements are the elements that have a high priority in the integration into the ontology for purpose fulfillment, and the rest can be discarded. The ontological form for candidate elements can be identified by analyzing their association concerning the rest of the components. Then, evaluate if the key elements will be used only for the ontology population or will impact the structure and the advantages and disadvantages that this could cause.

The candidate elements for the ontology network are the diseases and symptoms. They will continue to be represented in the form of individuals according to the structure of the ontology network. Their new relations will be represented as object properties and the clinical signs from the medical note as data-type properties. The effects of the drugs will be represented as instances according to their classification. Object properties will link them to the drugs according to their type (generalities, interactions, indications, among others).

#### Candidate element extraction

Once the candidate elements have been defined, the semi-automatic extraction must be carried out according to the following series of steps:

1. Tag Assignment: Each term (composed of one or more words) gets a syntactic or semantic tag in this step. The syntactic tags are assigned according to the grammatical features of each word. In contrast, for the semantic tag assignment, the terms are searched by similarity into external vocabularies. Whether they are found, acquiring the label provided by the corresponded vocabulary (e.g., Diabetes Mellitus is tagged as Disease according to the ICD-10 vocabulary). The vocabularies used for tag assignment medical note information are the International Classification of Diseases (ICD-10), a list of body parts (anatomy), signs, symptoms, and active pharmaceutical ingredients.
2. Nominal Phrases Identification: for terms that were only tagged syntactically, they are processed through syntactic patterns according to the language in order to find semantic entities free of an established vocabulary.
3. Triplet Formation: For this step, it is necessary to determine each triplet component, either by a NominalPhrase-Verb-NominalPhrase pattern or by establishing the domain through individuals previously inserted in the ontology (Individual-Verb-NominalPhrase).
  The relation identification in medical notes is through a set of patterns related to each type of tag. In the case of clinical signs, due to their definition that a clinical sign is a measurable manifestation, they will be taken as data-type properties by taking the verb has and adding the name of the clinical sign, and having as a range a type value chain or floating. For the rest, there is an implicit pattern within the medical notes, where there only exists a list of symptoms and diseases without a verb, they will be proposed as the range of the relations *presentsDisease* or *presentsSymptom* taking the Medical Note class as the domain.

#### Candidate triplets to ontological resources transformation

Once the candidate triples have been identified, the information is displayed on a control screen for the user, consisting of three fields (domain, relation, and range) containing the information of each element with a proposed IRI from the suggestion of the belonging domain. The user can modify this information to provide further certainty that the integration is correct; in this case, it is enough to press the *Register* button to continue with the process of modifying the involved ontologies or press the *Skip* button to discard the candidate triplet.

All triples identified as object properties from medical notes will be assigned with the IRI corresponding to the ontology network. In contrast, the data-type properties will be assigned within the Clinical Information Administration ontology.

#### Ontological resource integration

The integration of resources already with an assigned IRI begins through an information flow, which processes one element at a time. First, when receiving the IRI of the domain of the property, it verifies if it already exists within the ontology. If the resource exists, it is stored, and the process continues with the same analysis for the range. Otherwise, the incoming IRI is divided into two segments, identifying the name of the new resource and the IRI of the ontology to which it will be integrated. Then, the resource becomes an individual asserted into master class from the indicated ontology, and the original IRI is verified again. This same process is done if the range element does not exist in the ontology.

The same analysis is made using the IRI to verify its existence for the object properties. If it does not exist, the new property takes the suggested name and the domain and ranges information from the triplet; then, it is asserted with the IRI from the network and linked with the corresponding domain and range individuals.

### Step 6: Evaluation

An ontology network may be evaluated from the ontology modules that compose it plus some criteria for evaluating the connectivity and consistency composition of the network [50]. In this methodology, the Ontology Network evaluation is based on the quality criteria: satisfiability and consistency. The satisfiability is checked by the assertion of instances on each ontology and into the network, and the consistency is verified by the reasoner. Additionally, the competency of the ontology network is checked by the answers to the competency question.

## Results

This section describes the meta-relations used to unite the participating ontologies of the network, which are transformed into object properties using the IRI http://www.diabetes-mexico.org/red#. Nine meta-relations were obtained from meta-relation design stage and five more from non-ontological integration stage. All meta-relations have been implemented as object properties (see Fig. 2) with a domain and a range from different ontologies (see Table 4), also these are used to define some non-primitive classes described in Table 5. In general, the ontology network is composed of 1367 classes, 20 object properties, 63 data-type properties, and 4268 individuals from seven different ontologies.

**Table 4:** Domains and ranges of the meta-relations in the ontology network

Meta-relation	Domain	Range
isWrittenBy	Medical Note (Clinical Information Administration)	Doctor (Person)
presentsDisease	Medical Note (Clinical Information Administration)	Disease (Clinical Entity)
presentsSymptom	Medical Note (Clinical Information Administration)	Symptom (Clinical Entity)
writesMedicalNote	Doctor (Person)	Medical Note (Clinical Information Administration)
livesIn	Patient (Person)	Geographic Location (Geographic Location)
hasAPIInPrescription	Prescription Dose	ATC_Classification (Drug-ATC)
hasContraindication	Drug (Drug-ATC)	Clinical Entity (Clinical Entity)
hasDiagnosis	Medical Note (Clinical Information Administration)	Disease (Clinical Entity)
hasDisability	Clinical History (Clinical Information Administration)	Disability (Clinical Entity)
hasSideEffect	Drug (Drug-ATC)	Clinical Entity (Clinical Entity)
hasEducationLevel	Patient (Person)	Education Level (Education Level)
hasClinicalRecord	Patient (Person)	Clinical Record (Clinical Information Administration)
hasCaution	Drug (Drug-ATC)	Clinical Entity (Clinical Entity)
hasTreatmento	Medical Note (Clinical Information Administration)	Prescription Dose

**Table 5:** Non-primitive classes

Class	IRI	Description
Patient	http://www.personas-mexico. org/persona#Paciente	(livesIn exactly 1 ‘Geographic Location’) and (hasEducationLevel exactly 1 ‘Education Level’) and (hasClinicalRecord exactly 1 ‘Clinical Record’) and (tieneFechaNacimiento exactly 1 xsd:date) and (tieneSexo exactly 1 xsd:string)
Medical Note	http://www.modelo.org/ datos#Nota_Medica	(hasTreatment some ‘Prescription Dose’) and (hasHumanBiotype some ‘Human Biotype’) and (hasDiagnosis min 1 ‘Clinical Entity’) and (isWrittenBy exactly 1 ‘Doctor’) and (tieneGlucosaPostPrandial some xsd:integer) and (tieneAnalisis exactly 1 xsd:string) and (tieneExploracionFisica exactly 1 xsd:string) and (tieneFecha exactly 1 xsd:date) and (tieneFrecuenciaCardiaca exactly 1 xsd:integer) and (tieneFrecuenciaRespiratoria exactly 1 xsd:integer) and (tieneICC exactly 1 xsd:float) and (tieneIMC exactly 1 xsd:float) and (tienePadecimientoActual exactly 1 xsd:string) and (tienePeso exactly 1 xsd:float) and (tieneTMB exactly 1 xsd:float) and (tieneTalla exactly 1 xsd:float) and (tieneTemperaturaCorporal exactly 1 xsd:float)
Doctor	http://www.personas-mexico. org/persona#Medico	writesMedicalNote some ‘Medical Note’
Prescription Dose	http: //www.diabetes-mexico.org/ red#Dosis_En_Receta	(hasAPIPrescription some ‘Pharmaceutical Treatment’) and (tieneCantidadIndicadaEnReceta some xsd:float) and (tieneFrecuenciaIndicadaEnReceta some xsd:float) and (tieneMedidaIndicadaEnReceta some xsd:string)
Clinical History	http://www.modelo.org/datos#\\Historia_Clinica	hasDisability some ‘Disability’

The network was written in *Resource Data Framework* (RDF) language and was enriched by some axioms *Semantic Web Rule Language* (SWRL) in order to complement the representation with reasoning functions within the network. The functions are related to the calculation of the Body Mass Index (BMI), the human biotype through the BMI, the Waist-hip index, and the basal metabolic rate; some of them are described in Table 6.

**Table 6:** SWRL functions

Function	SWRL Description
BodyMassIndex	datos:hasWeight(?n, ?p) $\hat {}$ datos:hasHeight(?n, ?t)$\hat {}$ swrlb:multiply(?t2, ?t, ?t) $\hat {}$ swrlb:divide(?imc, ?p, ?t2) - > datos:hasBMI(?n, ?imc)
Waist-hip Index	datos:hasWaistMeasure(?n, ?ci) $\hat {}$ datos:hasHipMeasure(?n, ?ca) $\hat {}$ swrlb:divide(?icc, ?ci, ?ca) - > datos:hasWHI(?n, ?icc)
Overweight Function	datos:hasBMI(?n, ?imc) $\hat {}$ swrlb:greaterThanOrEqual(?imc, 25) $\hat {}$ swrlb:lessThan(?imc, 30) - > datos:hasHumanBiotype(?n, datos:Overweight)
Basal Metabolic Rate for women over 60 years old	persona:hasGender(?pa, ?sx) $\hat {}$ swrlb:stringEqualIgnoreCase(?sx, “Female”^^sxd:string) $\hat {}$ red:hasClinicalRecord(?pa, ?ec) $\hat {}$ datos:hasMedicalNote(?ec, ?nm) $\hat {}$ datos:hasAge(?nm, ?e) $\hat {}$ swrlb:greaterThanOrEqual(?e, 61) $\hat {}$ datos:hasWeight(?nm, ?p) $\hat {}$ swrlb:multiply(?aux, 10.5, ?p) $\hat {}$ swrlb:add(?tasa, ?aux, 596) - > datos:hasBMR(?nm, ?tasa)

**Fig. 3:**
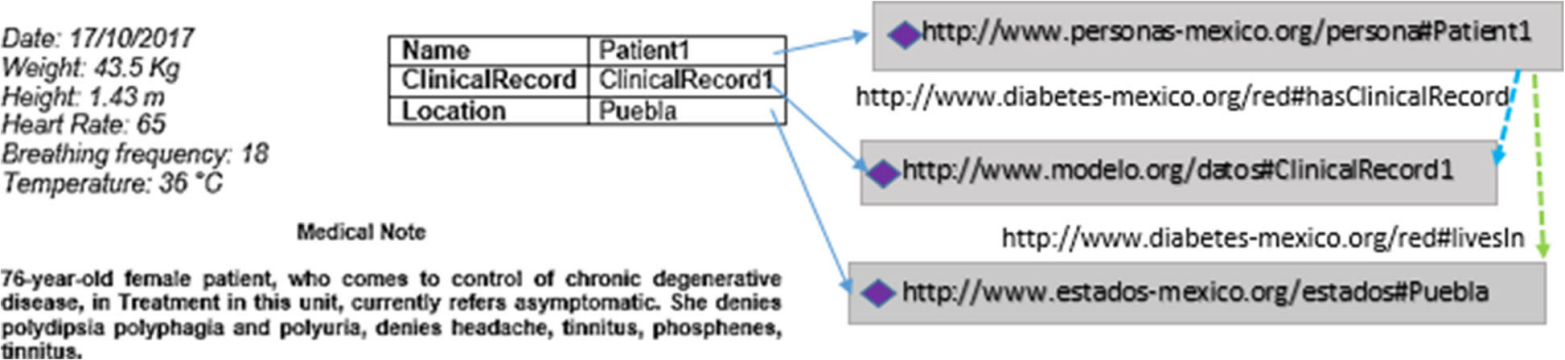
Object Property Assertions. Object creating from non-ontological resources using the IRI from Patient, Clinical Record, and Geographic Location. The arrows show the assertion of the livesIn and hasClinicalRecord object properties. These properties use the IRI from the Ontology Network

The integration of non-ontological resources within the network was used for two tasks: instance creation for satisfying the meta-relations of the network and ontology network expanding. In the former, the information from clinical records was classified according to the IRI of each domain. For the object properties assertions between domains, the IRI of the network has been used. Figure 3 shows an example of objects from a clinical record and the use of the IRI according to their ontological classification. This makes it possible to check the model satisfiability by verifying that there are instances for each of the participating ontologies of the network. During the population process based on the information structure for creating new instances of each of the ontologies and the network, a total of 6,275 individuals and 3,519 anonymous nodes belonging to the Drug-ATC ontology and the network were identified. The total of instances per ontology are shown in Table 7.

**Table 7:** Instances per ontologies

Ontology	Individuals
Person	171
Clinical Information Administration	1090
Clinical Entity	271
Geographic Location	32
Education Level	13
Control Plan	577

In the ontology network expanding task, five new meta-relations as object properties were added; Table 8 shows the domains, ranges, and the number of insertions related to each of them.

**Table 8:** Ontology network expanding results

Object Property	Domain	Range	Assertions
presentsDisease	Medical Note	Disease	377
presentsSymptom	Medical Note	Symptom	6
hasSideEffect	Drug	Clinical Entity	297
hasContraindication	Drug	Clinical Entity	176
hasCaution	Drug	Clinical Entity	27

**Fig. 4:**
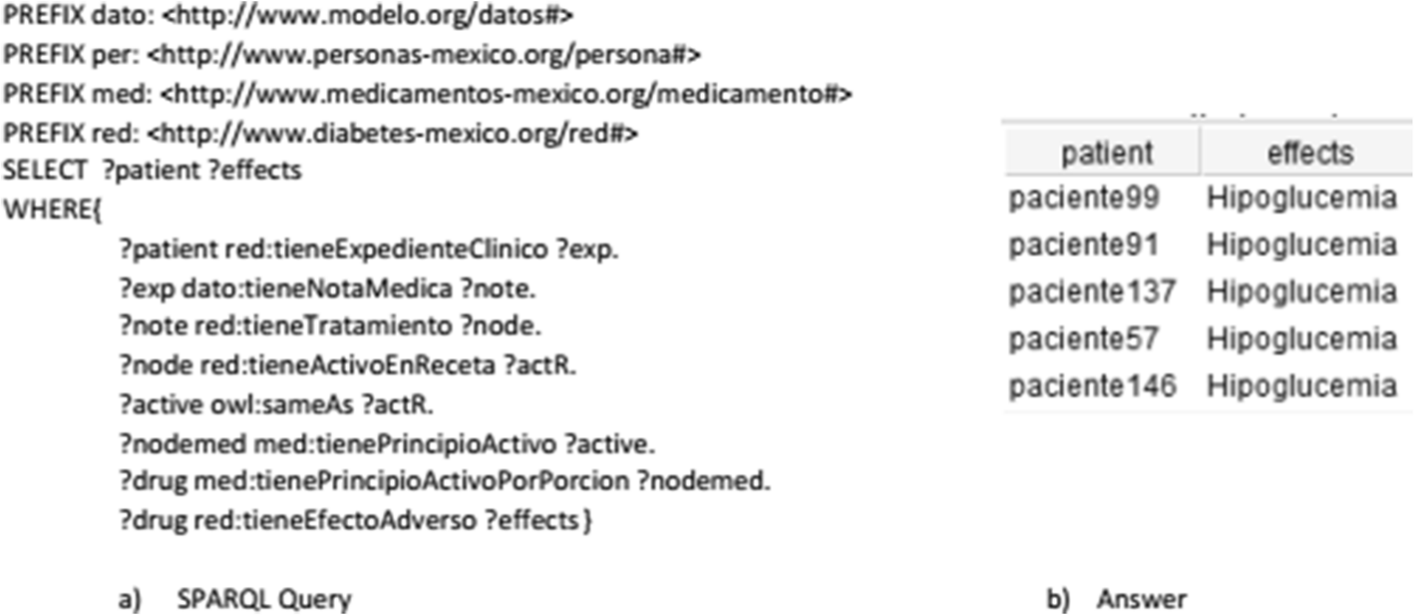
Side Effects of a Drug in a Patient. SPARQL query and its answer about the side effects that a patient can experience respect it pharmacologic treatment

Another impact of integrating non-ontological resources to the ontology network is the ability to make more specialized queries and generating new inferences through SWRL rules considering the aggregated data. An example of this is the query about side effects that a patient may have regarding the drug treatment indicated in the medical note (see Fig. 4). This example works through a rule that generates an alert to warn about a contraindicated drug regarding the diagnosis given in the same medical note.

**Fig. 5:**
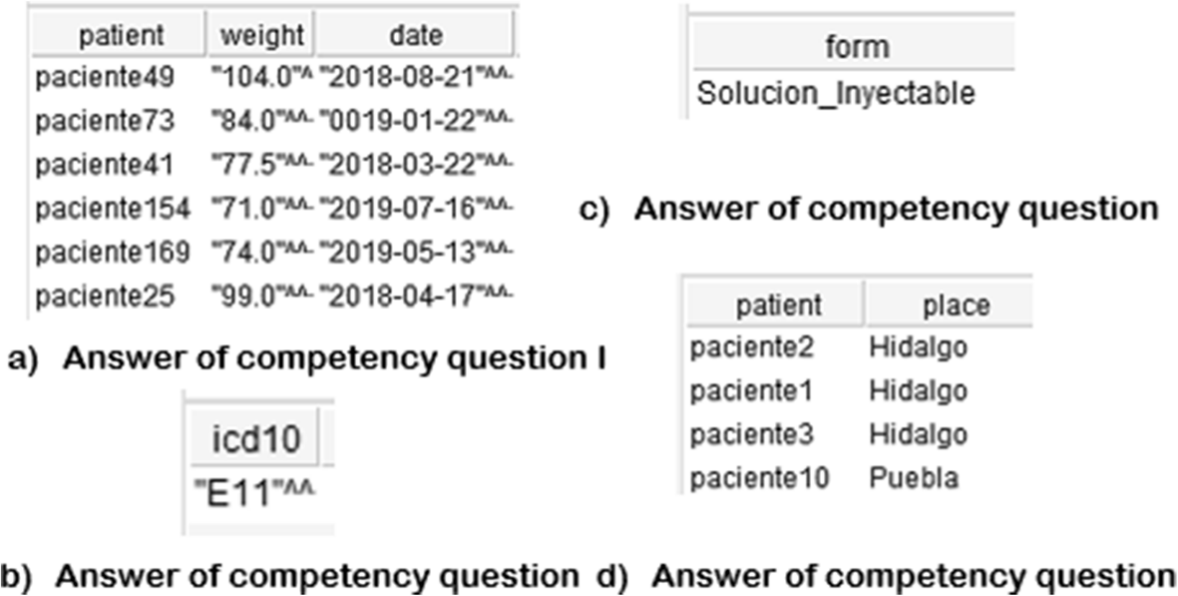
Competency Questions Answers. These answers are some of the answers used for ontology network evaluation

The resulting Ontology Network was evaluated by verifying the fulfillment of the purpose for which it was designed. The purpose of the ontology was expressed by general, and specific-domain competency questions, which were translated to *SPARQL* queries for evaluation. *SPARQL* is a lightweight, triple-based query language that makes it easy to query the ontology network. Translations of some competency queries in SPARQL are shown in Table 9. Another important evaluation criterion is the verification of the logical consistency of the Ontology Network; therefore, additional logic-based reasoners such as Pellet and Drools were used since they support SWRL inference rules, obtaining favorable results (see Fig. 5).

**Table 9:** Competency questions from ontology network requirements

Competency Question	SPARQL Query	Pseudo-code
I) What is the weight of a patient?	PREFIX dato: <http://www.modelo.org/datos# >	List all triplets *(?patient, ?weight, ?date)*
	PREFIX red: <http://www.diabetes-mexico.org/red# >	Where for the ?patient has a clinical record
	SELECT ?patient ?weight ?date ?x	?record (via *dato:tieneExpedienteClinico*),
	WHERE { ?patient red:tieneExpedienteClinico ?record.	?record has a medical note ?note (via
	?record dato:tieneNotaMedica ?note.	*dato:tieneNotaMedica*), ?note contains the
	?note dato:tienePeso ?weight.	wieght ?weight (via *dato:tienePeso*) and a
	?note dato:tieneFecha ?date}	registration date ?date (via *dato:tieneFecha*)
II) What is the ICD-10 code for T2DM?	PREFIX enf: <http://www.padecimientos-mexico.org/enfermedad# >	List the ICD-10 code ?icd10 of the T2DM individual linked via an annotation property
	PREFIX red: <http://www.diabetes-mexico.org/red# >	(*red:ICD10*).
	SELECT ?icd10	
	WHERE { enf:DiabetesMellitusTipo2 red:ICD10 ?icd10}	
III) What are the presentations of Insulin Lispro?	PREFIX medi: <http://www.medicamentos-mexico.org/medicamento# >	List the different pharmaceutical forms ?form of the drugs ?x (via
	SELECT distinct ?form	*medi:tieneFormaFarmaceutica*) that
	WHERE { ?x medi:tienePrincipioActivo medi:Insulina_Lispro.	have the Insulin Lispro drug individual
	?y medi:tienePrincipioActivoPorPorcion ?x.	(*medi:Insulina_Lispro*) as API (via
	?y medi:tieneFormaFarmaceutica ?form}	*medi:tienePrincipioActivo*)
IV) Where the patient *X* is living?	PREFIX red: <http://www.diabetes-mexico.org/red# >	List all pairs *(?patient, ?place)* Where for the
	SELECT ?patient ?place	?patient the ?place is linked to the ?patient
	WHERE { ?patient red:resideEn ?place}	(via “red:resideEn”)

Once the competency-based evaluation of the network was completed, the search for pitfalls was carried out through the OOPS tool [51] in order to make an external evaluation of the Ontology Network design. The following remarks were obtained: found some elements lacking a description through metadata tagged by *rdfs:label* or *rdfs:comment* despite having them; they indicate the lack of inverse relations. However, these relations are not considered essential within the network, and placing them would mean an increase in computational cost that would be unnecessary. The evaluation also indicates different naming conventions within the network due to different ontologies, as minor relevance pitfalls. Also, important pitfalls were found, such as the absence of the participation of elements as the SWRL prefixes in non-primitive classes definition. However, SWRL prefixes within the network are not focused on the classification but the assertion of object properties.

Another evaluation aspect applied to the network was to answer some competency questions analyzed in [52], these questions have been endorsed in [53] and have been selected since they were designed for an ontology of the medical domain and can be answered through the network. Table 10 shows the external competency questions and their SPARQL translation.

**Table 10:** External competency questions

Competency Question	SPARQL Query	Pseudo-code
What are the main education levels?	PREFIX edu: <http://www.niveleseducativos-mexico.org/niveles# > PREFIX rdfs: <http://www.w3.org/2000/01/rdf-schema# >	List all the individuals ?x that belonging to each subclass of Education Level (*edu:Escolaridad*)
	SELECT ?x	
	WHERE {?ec rdfs:subClassOf edu:Escolaridad.	
	?x a ?ec}	
What are the main categories a person may belong to?	PREFIX rdfs: <http://www.w3.org/2000/01/rdf-schema# > PREFIX per: <http://www.personas-mexico.org/persona# > SELECT ?category	List the categories ?category in which the Person class (*per:Persona*) is divided (via *rdfs:subClassOf*)
	WHERE {?category rdfs:subClassOf per:Persona}	
What types of data are collected during clinical consultation?	PREFIX dato: <http: //www.modelo.org/datos# > PREFIX rdfs: <http: //www.w3.org/2000/01/rdf-schema# > SELECT ?data ?range	List the data ?data and the type-data ?range that compose (via rdfs:range) the medical note (*dato:Nota_Medica*)
	WHERE { ?data rdfs:domain dato:Nota_Medica. ?data rdfs:range ?range}	
What are the types of diagnosis?	PREFIX rdfs: <http://www.w3.org/2000/01/rdf-schema# > PREFIX enf: <http://www.padecimientos-mexico.org/enfermedad# > SELECT ?diagnosis	List the diagnosis classification ?diagnosis in which is divided (rdf:subClassOf) the Clinical Entity class (*enf:Enfermedad_Clinica*)
	WHERE { ?diagnosis rdfs:subClassOf enf:Entidad_Clinica}	

## Discussion

In this work, an ontology network has been built from six well-defined domains (Control Plan, Clinical Entity, Education Level, Clinical Information Administration, Geographic Location, and Person), resulting in new classes, data properties, meta-relations, and axioms for their interconnection.

The development of an Ontology Network about DM allows highlighting the features of the domain and the identification of the participating pieces of the domains. This allows having small ontological models that provide more readability of all the participating elements and the opportunity to enrich or use them independently.

One benefit of using a network approach for constructing an ontological model with different domains is to facilitate the maintenance of each ontology without compromising the resources of the others; that is, each domain can be modified or updated without affecting the final structure of the network.

The Ontology Network can be used in clinical consultation since it has the patient’s basic information and additional information that can help the doctor make decisions regarding the diagnosis and treatment of the disease. In addition, the network can be used in the teaching process since the structure lends itself to capturing and managing the knowledge of the doctor acquired through experience, thus facilitating the transmission of the same.

The resolution of the competency questions through SPARQL language allows querying any feature of the ontology network, including information about its structure. Another alternative for querying the ontology network is to use DL-Query; however, with DL-Query, it is not possible to obtain specific values from literals easily.

## Conclusions

In this work, an Ontology Network about the DM in Mexico was created using ontological and non-ontological resources in order to offer a semantic structure for assisting in the medical diagnosis process. The composition of the network provides a set of well-defined ontological modules facilitating the reuse of one or more of them. The inclusion of international vocabularies as SNOMED CT or ICD-10 reinforces the representation by international standards. It increases the semantic interoperability of the network, providing the opportunity to integrate other ontologies with the same vocabularies. The ontology network design methodology offers a guide for ontology developers about how to use ontological and non-ontological resources in order to exploit the maximum of information and knowledge from a set of domains that share or not information.

This network is composed of well-defined modules because it is easily reusable to integrate information about patients suffering from different diseases than diabetes since the information from multiple domains is contemplated, which could be linked through new meta-relations and rules of inference that favor the representation of another disease.

For future work, other meta-relations will be added in order to design new alerts about the possible complications related with values of clinical signs such as high glucose or cholesterol values. Additional domains such as anatomy will be considered for the integration of more candidate triplets that help to identify with more certainty the clinical entities.

## Data Availability

Data sharing is not applicable for this article, as no data sets were generated or analyzed during its creation. The Ontology Network about the DM in Mexico and the related ontologies are available in http://caliope.cs.buap.mx/IngenieriaOntologica/recursos.html

## Abbreviations

API: Active Pharmaceutical Ingredient
ATC: Anatomical Therapeutic Chemical classification system
DDR: DM-Disease Relation
DL-Query: Description Logic Query language
DM: Diabetes Mellitus
FH: Family History
ICD-10: International Classification of Disease Tenth Revision
IRI: Internationalized Resource Identifier
OOPS: Ontology Pitfall Scanner
PD: Personal Data
RDF: Resource Data Framework
SCTID: SNOMED CT Identifier
SNOMED CT: Systematized Nomenclature of Medicine-Clinical Terms
SR: Semantic Rules
SWRL: Semantic Web Rule Language
Tt: Treatment
T1DM: Type 1 Diabetes Mellitus
T2DM: Type 2 Diabetes Mellitus

## Footnotes

1 https://hup.buap.mx/
